# The Angiogenic Potential of Mesenchymal Stem Cells from the Hair Follicle Outer Root Sheath

**DOI:** 10.3390/jcm10050911

**Published:** 2021-02-26

**Authors:** Vuk Savkovic, Hanluo Li, Danilo Obradovic, Federica Francesca Masieri, Alexander K. Bartella, Rüdiger Zimmerer, Jan-Christoph Simon, Christian Etz, Bernd Lethaus

**Affiliations:** 1 Department of Oral and Maxillofacial Surgery, Leipzig University Medical Center, 04103 Leipzig, Germany; Vuk.Savkovic@medizin.uni-leipzig.de (V.S.); hanluo.li@medizin.uni-leipzig.de (H.L.); Alexander.Bartella@medizin.uni-leipzig.de (A.K.B.); Ruediger.Zimmerer@medizin.uni-leipzig.de (R.Z.); 2 Department of Cardiology and Angiology, Leipzig Heart Center, 04289 Leipzig, Germany; daniloobradovic2@gmail.com (D.O.); christian.etz@medizin.uni-leipzig.de (C.E.); 3 Biotechnology Unit, Life Sciences, School of (EAST) Engineering, Arts, Science & Technology, University of Suffolk, Ipswich IP41QJ, UK; F.Masieri@uos.ac.uk; 4 Department of Dermatology, Venereology and Allergology, Leipzig University Medical Center, 04103 Leipzig, Germany; Jan-Christoph.Simon@medizin.uni-leipzig.de

**Keywords:** MSC, mesenchymal stem cells from hair follicle outer root sheath, MSCORS, neoangiogenesis, mesenchymal stem cells, vascularization, tissue-engineering

## Abstract

Neovascularization is regarded as a pre-requisite in successful tissue grafting of both hard and soft tissues alike. This study considers mesenchymal stem cells from hair follicle outer root sheath (MSCORS) as powerful tools with a neat angiogenic potential that could in the future have wide scopes of neo-angiogenesis and tissue engineering. Autologous MSCORS were obtained ex vivo by non-invasive plucking of hair and they were differentiated in vitro into both endothelial cells and vascular smooth muscle cells (SMCs), two crucial cellular components of vascular grafts. Assessment was carried out by immunostaining, confocal laser-scanning microscopy, gene expression analysis (qRT-PCR), quantitative analysis of anastomotic network parameters, and cumulative length quantification of immunostained α-smooth muscle actin-containing stress fibers (α -SMA). In comparison to adipose mesenchymal stem cells, MSCORS exhibited a significantly higher differentiation efficiency according to key quantitative criteria and their endothelial derivatives demonstrated a higher angiogenic potential. Furthermore, the cells were capable of depositing their own extracellular matrix in vitro in the form of a membrane-cell sheet, serving as a base for viable co-culture of endothelial cells and SMCs integrated with their autologous matrix. Differentiated MSCORS hereby provided a complex autologous cell-matrix construct that demonstrates vascularization capacity and can serve as a base for personalized repair grafting applications.

## 1. Introduction

Angiogenesis is an essential process in embryonic development as well as in postnatal tissue formation and regeneration. Initiated by endothelial cells, angiogenesis is followed by vascular remodeling and blood vessel maturation, in synergy with vascular smooth muscle cells (SMCs) [1]. As such, endothelial cells and SMCs are the two inevitable cellular components for successful vascularization and efficient regenerative therapies. The strategies addressing perfusion to optimize vascularization after grafting, including vascularization of bone grafts, mostly rely on angiogenic growth factor delivery, vascular cell implantation, gene therapy and biomaterial engineering [2]. Among these options, the combined delivery of stem cells and growth factors, especially pro-angiogenic factors, has been pinpointed as particularly efficient for recapitulating the physiological process of vascularization [2,3]. These processes are vital in the regeneration of any tissue regeneration, including bone.

Various sources of cells have been employed in regeneration and reconstruction of small blood vessels, including endothelial cells, SMCs, and/or fibroblasts and mesenchymal stem cells (MSCs) [4,5,6,7,8]. In particular, MSCs qualify as excellent “seed cells” candidates due to their potential to differentiate also into endothelial and smooth muscle cells, together with their immune-privileged status and paracrine effects [9,10]. Nevertheless, MSCs represent valid allies in the regeneration of cartilage [11] and in the effective engineering of a plethora of soft tissues, particularly in fat grafting [12]. Notably, MSCs have also crucial roles in osteogenesis and clinical bone repair applications, which are particularly challenging [2,13,14], especially in femur head engineering [15] or oral rehabilitation [16,17,18], amongst others.

One of the key issues in obtaining MSCs is that harvesting is often combined with various degrees of donor site morbidity. MSCs have previously been isolated from hair follicle outer root sheath [19,20,21]. Among the non-invasively available autologous sources of MSCs, human hair follicle outer root sheath (ORS) has emerged as one of the easiest sources with the most putative output, hereby superseding the former attempts [22,23]. These cells, named mesenchymal stem cells from the outer root sheath (MSCORS) previously demonstrated for a scalable cell proliferation and a differentiation potential towards osteogenic, adipogenic and chondrogenic lineages that was at least comparable to that of bone marrow- and adipose tissue-derived MSCs (BMMSC and ADMSC, respectively). Together with tri- mesoderm lineage differentiation, induction towards endothelial cell and smooth muscle cell (SMC) lineages were also addressed, to exert their angiogenic capacity [23].

In this study, we further elaborated the capability of MSCORS to give rise to endothelial cells and smooth muscle cells for possible angiogenic applications. We compared their explicit angiogenic networking capability with that of ADMSCs as an established control group. Another objective was to establish and evaluate a co-culture of the endothelial and smooth muscle lineage in an autologous extracellular matrix that could provide a useful proof of concept for future tissue engineering applications.

## 2. Materials and Methods

Hair follicles were collected from healthy donors (*n* = 7) within the age range of 25–45 years. Adipose tissue (0.5–5.8 g/patient) was obtained from healthy patients (*n* = 5; 2 men, 3 women) within the age range of 23–54 years who underwent general trauma or orthopedic surgery. Experiments were performed 3 times with 3 experimental repetitions.

### 2.1. Isolation and Differentiation of MSCORS and Adipose-Derived MSCs (ADMSCs)

MSCORS from human hair follicles were obtained using a non-invasive method of air–liquid interface, as previously described [23]. Briefly, anagen human hair follicles (n = 30–50) were non-invasively plucked from donor’s occipital region and rinsed in MSC Washing Medium (Table S1). Hair shafts were shortened and the proximal dermal papilla was excised; then the hair follicles were extensively washed, digested using 5 mg/mL collagenase X (Sigma-Aldrich GmbH, Schnelldorf, Germany) for 12 min, and seeded onto the 0.4 μm porous Transwell membrane (Corning Inc., New York, NY, USA). The lower chamber of the Transwell was filled with MSC Isolation Medium (Table S1). Cell culturing was carried out under hypoxic conditions (5% O_2_, 5% CO_2_) at 37 °C for 21 days. Medium was changed twice a week. After ORS cells migrated out of the hair follicle and formed a layer, these were detached using 0.04%/0.03% Trypsin/EDTA (PromoCell GmbH, Heidelberg, Germany) into a single-cell suspension and subcultured onto a 6-well plate. After a 48 h attachment period, the cells were cultured further and passaged at 90% confluence. The cells between p1 and p5 were used for further assessments.

Adipose tissue was thoroughly rinsed using MSC Washing Medium (Table S1), sliced into 8 mm^3^ pieces, and digested in 2 mg/mL collagenase X (Sigma-Aldrich GmbH, Schnelldorf, Germany) at 37 °C for 4 h. After neutralization with FBS, the digestion mix was vigorously vortexed and centrifuged at 600× *g* for 10 min at room temperature. The pellet was re-suspended, washed with DPBS, filtered through a 100 μm nylon strainer and seeded onto a 75 cm^2^ cell flask in MSC Cultivation Medium (Table S1). The attached and proliferating cells were cultured in hypoxic conditions (5% O_2_, 5% CO_2_) at 37 °C with two medium changes per week and subcultured at 90% confluence.

Differentiation towards endothelial and smooth muscle tissue was induced according to given protocols and media (Table S1) [23]. MSCORS and ADMSC harvested before passage 5 were seeded at a density of 1.5 × 10^4^ cells/cm^2^. Their differentiation was carried out by exposure to 10 ng/mL TGF-β1, using human aortic smooth muscle cells (HAoSMCs) as control. Differentiation towards endothelial lineage was induced in cells seeded at density of 2.5 × 10^4^ cells/cm^2^ with 5 ng/mL bone morphogenic factor 4 (BMP4) and 30 ng/mL vascular endothelial growth factor (VEGF), using native human umbilical vein endothelial cells (HUVEC) as control. After 21 days and 28 days of differentiation, endothelial cells were dissociated into single cells using Trypsin/EDTA, and proceeded to qRT-PCR, immunostaining and tube-forming assay. Differentiation into smooth muscle cells was analyzed at the level of protein expression by immunostaining of alpha smooth muscle actin (αSMA). Moreover, following cell dissociation using Trypsin/EDTA αSMA gene expression was characterized by the means of qRT-PCR.

### 2.2. Tube-Forming Assay

To assess the endothelial differentiation of MSCs/ADMSCs and evaluate their potential to form anastomoses, tube-forming assay was performed using Corning^®^ Matrigel^®^ Matrix (Corning Inc., Lowell, MA, USA) as previously described [23]. Briefly, Matrigel was carefully added to the bottom of a 48-well plate, and the differentiated endothelial cells were dissociated and seeded onto Matrigel membrane at a density of 6 × 10^4^ cells per well in Endothelial Medium (Table S1). The assay was incubated for 8 h in hypoxic conditions with 5% O_2_ and 5% CO_2_ at 37 °C. The resulting angiogenic tubules formed by differentiated endothelial cells were stained with Live/Dead Assay (Calcein AM/Propidium Iodide, PI; ThermoFisher Scientific Inc., Waltham, MA, USA). The angiogenic anastomotic network with live/dead fluorescence was imaged by Keyence Fluorescence Microscope.

### 2.3. Quantitative ImageJ Analysis for Angiogenic Assay

The inherent ability of endothelial cells to form anastomotic interconnected tubules was quantified by measuring the attributes of the network formed. By using software ImageJ and the Angiogenesis Analyzer plug-in tool, several indicators of tube-forming elements were analyzed, including number of Junctions, Segments, Branches, Meshes, Master Junctions, Master Segments and Total Branching Length, as previously described [24].

ImageJ plug-in Angiogenesis Analyzer allowed a quantitative evaluation of the vessel-like network organization. To better understand various indicators of in vitro pseudo capillary structure, the terminology of different parameters with detailed explanation is presented as follows, based on the provided instruction [24].

A segment of angiogenic tube with a free-ending on one side, and the other end connected to a junction point, hereby forms a structure resembling a “Branch”. “Segments” represent portions of an angiogenic capillary with two ends connected to two junction points. If these two junctions are “Master Junctions”, this segment is called a “Master Segment”. “Junctions” indicate the multi-intersection junctions with three or more furcated branches in the angiogenic structures. If furcated branches of the junction are exclusively connected with other capillary structures and thereby without extremities, this junction is called a “Master Junction”. “Meshes” are closed areas formed by segments. “Total branching length” is the total length of all recognizable capillary tubes in the analyzed area, including all the segments and branches. “Total branching length” and “Total Segment Length” were analyzed and calculated.

### 2.4. Immunostaining

We performed Immunofluorescence staining according to the following protocols, as previously reported [23]. Briefly, differentiated cells were detached from the flask, seeded onto Falcon™ Chambered Cell Culture Slides (ThermoFisher Scientific Inc., Waltham, MA, USA), and fixed with 4% Paraformaldehyde (PFA) for 12 min. After DPBS washing, cells were blocked with 10% Normal Goat Serum (Sigma-Aldrich GmbH, Schnelldorf, Germany) for 20 min, and incubated with primary antibody targeting endothelial marker CD31 (mIgG1, Clone P2B1, 1:100 dilution, Abcam Plc, Cambridge, MA, USA) and smooth muscle marker αSMA (mIgG1, Clone 1A4, 1:100 dilution, Abcam Plc, Cambridge, MA, USA). After three washes using DPBST (0.5% Tween 20 in DPBS, Table S1), goat anti-Mouse IgG conjugated with Alexa Fluor 594 secondary antibody (1:400 dilution, ThermoFisher Scientific Inc., Waltham, MA, USA) was applied. Nuclei were labeled with 4′, 6-diamidino-2-phenylindole (DAPI, 1:500 dilution, ThermoFisher Scientific Inc., Waltham, MA, USA). Cells on chamber slides were mounted and imaged using Keyence BZ-9000 Fluorescence Microscope (Keyence GmbH, Neu-Isenburg, Germany) or LSM700 confocal microscope (Carl Zeiss AG, Oberkochen, Germany).

### 2.5. Quantitative Analysisof the Length of Immunostained αSMA Fibers Using ImageJ software

To analyze the immunostained α-Smooth Muscle Actin (αSMA)-containing stress fibers, the software ImageJ was employed. For this purpose, the image of αSMA immunostaining was sharpened and converted to an 8-bit image. A threshold was adjusted to obtain clearly visualized filament structure of the αSMA fibers and reduce the signal background. The plug-in “Angiogenesis Analyzer” was used to recognize and mark the fibers’ pattern.

### 2.6. qRT-PCR to Determine Gene Expression of Endothelial and Smooth Muscle Cell Markers

The gene expressions of endothelial (*CD31*, *CD105*, Vascular Endothelial Growth Factor-*VEGF*, Von-Willebrand Factor-*VWF*) and smooth muscle markers (actin alpha 2*-ACTA2*, Tubulin Beta Chain*-TUBB*, Calponin 1*-CNN1*) were determined using qRT-PCR as previously described [23]. Briefly, differentiated MSCs were split using cell scraper and collected in Qiazol Lysis Reagent (Qiagen, Hilden, Germany). Total RNA was extracted using RNeasy Plus Universal Kit (Qiagen, Hilden, Germany) and quantified by Nanodrop 2000 spectrometer (ThermoScientific Inc., Waltham, MA, USA). An amount of 1 μg of mRNA was reverse-transcribed into cDNA using QuantiTect Reverse Transcription Kit (Qiagen, Hilden, Germany). qRT-PCR for targeted genes was performed using QuantiFast SYBR^®^ Green PCR Kit (Qiagen, Hilden, Germany). A total of 5–50 ng cDNA was used for a 20 μL reaction. Thermal cycling was set up at 95 °C for 60 s, followed by 40 cycles of 95 °C for 10 s, and 60 °C for 30 s. Gene expression levels were analyzed using 7500 Software v2.3 (ThermoFisher Scientific Inc., Waltham, MA, USA), normalized to housekeeping gene hypoxanthine-guanine phosphoribosyltransferase (HPRT), and calculated against the control represented by cDNA derived from undifferentiated MSCs using 2^−ΔΔCt^ method for relative quantification. Primers were designed using Primer3 web version 4.1.0 (60 °C annealing temperature) and manufactured by Invitrogen. The primer sequences are specified in Table S2. PCR amplification efficiency was determined using the method of 10-fold serial dilutions of cDNA, in terms of linear regression of the amplification on plotted logarithmic scale.

### 2.7. Production of MSCORS-SM-Sheet

To construct the MSCORS-comprised smooth muscle sheet (MSCORS-SM-Sheet) in form of a deposited extracellular matrix, a confluent culture of MSCORS was directed towards smooth muscle differentiation with a procedure in situ. Briefly, the MSCORS were seeded onto the 12 × 12 cm^2^ square petri dishes at cell seeding density of 1.5 × 10^4^ cells/cm^2^ in MSC Cultivation Medium (10% Fetal Bovine Serum, 2 mM L-Glutamine in DMEM (Low Glucose), Table S1). After 48 h, the medium was changed to MSC Smooth Muscle Medium (10 ng/mL TGFβ-1, 10% Fetal Bovine Serum, 2 mM L-Glutamine in Low Glucose DMEM, Table S1) with addition of 0.5 µM Sodium Ascorbate, and incubated for 4 weeks in hypoxic conditions (5% O_2_ and 5% CO_2_ at 37 °C). Medium was changed twice a week.

### 2.8. MSCORS-Derived Endothelial Cells Attachment to MSCORS-SM-Sheet

To study the attachment and interactions of endothelial cells and smooth muscle cell sheet, the MSCORS-derived endothelial cells and MSCORS-SM-Sheet were pre-labeled with different fluorescent dyes. Briefly, the MSCORS endothelial cells obtained from endothelial differentiation were dissociated into single cells and labeled with an aliphatic fluorescent dye PKH26 (PKH26 Red Fluorescent Cell Linker Mini Kit, Sigma-Aldrich Chemie GmbH, Steinheim, Germany at a concentration of 2 × 10^−6^ M for 30 min in hypoxic conditions (5% O_2_ and 5% CO_2_ at 37 °C). The cells in MSCORS-SM-Sheet were labeled with PKH67 fluorescent dye (PKH67 Green Fluorescent Cell Linker Mini Kit Sigma-Aldrich Chemie GmbH, Steinheim, Germany) by directly incubating with PKH67 at 2 × 10^−6^ M in hypoxic conditions (5% O_2_ and 5% CO_2_ at 37 °C) for 2 h. The labeled endothelial cells were seeded directly onto the MSCORS-SM-Sheet. After 24 h of cultivation, the membrane complex was examined and imaged by LSM 700 confocal microscope (Carl Zeiss AG, Oberkochen, Germany).

### 2.9. Statistical Analysis

All quantitative data were statistically analyzed using unpaired student’s t-test. Normal distribution was assessed by a Shapiro–Wilk normality test and Kolmogorov–Smirnov test. *p* values ≤ 0.05 were considered as statistically significant.

## 3. Results

### 3.1. Endothelial Differentiation of MSCs in 2D

**Figure 1:**
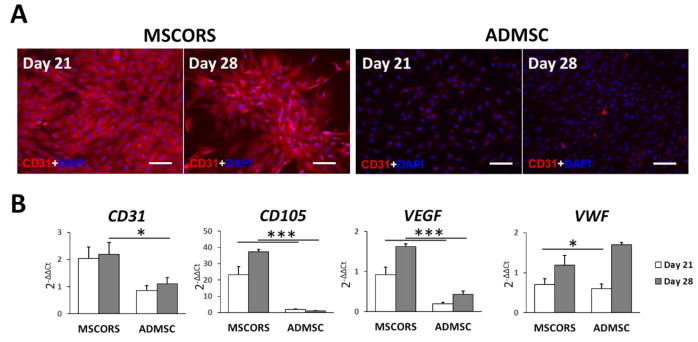
Endothelial differentiation of mesenchymal stem cells from the outer root sheath (MSCORS) and adipose-derived mesenchymal stem cells (ADMSC) in vitro. ADMSC and MSCORS were induced in vitro towards endothelial lineage for 21 and 28 days, and differentiation was evaluated by the means of immunostaining and qRT-PCR. (**A**) CD31 immunostaining on ADMSC and MSCORS (scale bar: 100 μm; magnification 20×). (**B**) Gene expression of endothelial markers in ADMSC and MSCORS during endothelial differentiation. Expression levels were normalized to the housekeeping gene HTRP-1 and calculated using 2^-ΔΔCt^ method against undifferentiated controls of ADMSC and MSCORS. Results are shown as mean ± SD. (* *p* < 0.05, *** *p* < 0.01).

Differentiation of MSCORS and ADMSC towards endothelial lineage was successfully induced by bone morphogenic factor 4 (BMP4) and vascular endothelial growth factor (VEGF*)*. The outcome of endothelial differentiation was confirmed by CD31 immunostaining as published before (Figure 1A) [23]. It was further assessed by the expression of endothelium-related genes *CD31*, *CD105*, Vascular Endothelial Growth Factor (*VEGF)*, and von Willebrand factor (*VWF*)*,* by the means of qRT-PCR (Figure 1B).

After 21 and 28 days of endothelial differentiation, visible cell morphology changes were observed at day 7 from the start of the differentiation induction. MSCORS and ADMSCs acquired a cobblestone-like shape with smaller size and closer cell–cell contacts, typical of endothelial cell morphology [25]. The size of the MSCORS- and ADMSC-differentiated endothelial cells was smaller compared to that of undifferentiated cells in the cell suspension (Figure S1B).

Intensive CD31 immunostaining with a clear membrane expression pattern was found in endothelial-differentiated MSCORS (Figure 1A) as opposed to that observed in ADMSCs. Nevertheless, MSCORS- and ADMSC-derived endothelial cell pattern of CD31 did not co-localize with the cell junctions to the same extent observed in native human umbilical vein endothelial cells (HUVEC) (Figure S1C). The undifferentiated controls of MSCORS and ADMSC did not express detectable levels of CD31 (Figure S1A).

The mRNA expression levels of endothelial cell differentiation marker genes *CD31*, *CD34*, *CD105*, *VEGF*, and *VWF* and their relative gene expression alteration following endothelial differentiation were calculated against undifferentiated control cells using 2^−ΔΔCt^ method. Expression levels and the increase in endothelial markers were higher in MSCORS than in ADMSC, for all assessed differentiation markers (Figure 1B).

### 3.2. Angiogenic Potential of Differentiated MSCORS and ADMSCs

To study the angiogenic capacity of endothelial cells differentiated from MSCORS and ADMSC, Matrigel-based tube-forming assay was employed. Following the assay, microphotographs were collected and analyzed quantitatively recording anastomotic network parameters utilizing ImageJ software.

**Figure 2:**
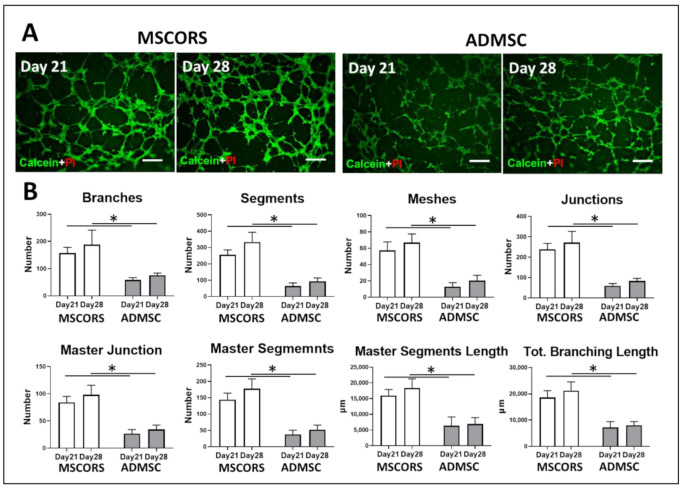
Tube-forming assay of endothelial differentiated mesenchymal stem cells from the outer root sheath (MSCORS) and adipose-derived mesenchymal stem cells (ADMSC) with quantitative analysis. The angiogenic functionality of the differentiated endothelial cells from MSCORS and ADMSC were investigated using tube-forming assay to assess the potential for anastomosis. (**A**) Microphotographs of anastomotic network generated by differentiated MSCORS and ADMSCs. Both MSCORS and ADMSC formed interconnected network of anastomotic tubes after endothelial differentiation, with living cells stained by green fluorescence and dead cells stained in red (scale bar 250 μm; magnification 4×). (**B**) Quantitative analysis of sprouting angiogenesis on Matrigel-based tube-forming assay using ImageJ Angiogenic Analyzer in the given parameters. Results are shown as mean ± SD. (* *p* < 0.05).

After 21 and 28 days of endothelial differentiation, tube-forming assay (Figure 2A) revealed that both differentiated MSCORS and ADMSCs formed a complex anastomosis network on the surface of Matrigel. Interestingly, the network formed by MSCORS was more complex and branched than that of ADMSCs. A majority of differentiated MSCORS cells constituted a network of interconnected capillaries with a very limited number of isolated cells. The complexity of the network formed by 28-day-differentiated MSCORS was higher than that of 21-day-differentiated MSCORS. Less ADMSCs were involved in forming the capillary networks, building fewer connections between tubes (Figure 2A). The angiogenesis network formed by endothelial cells differentiated from MSCORS showed a quantitatively more complex organization than that of ADMSCs, in terms of numbers of Junctions, Segments, Branches, Meshes, Master Junctions, Master Segments, Master Segments Length and Total Branching Length both at 21 and 28 days of endothelial differentiation (Figure 2B). Furthermore, MSCORS that underwent 21 and 28 days of endothelial differentiation showed an anastomosis capacity comparable to that of HUVECs (Figure S1C,D) and higher than ADMSCs.

### 3.3. Smooth Muscle Differentiation of MSCs in 2D

**Figure 3:**
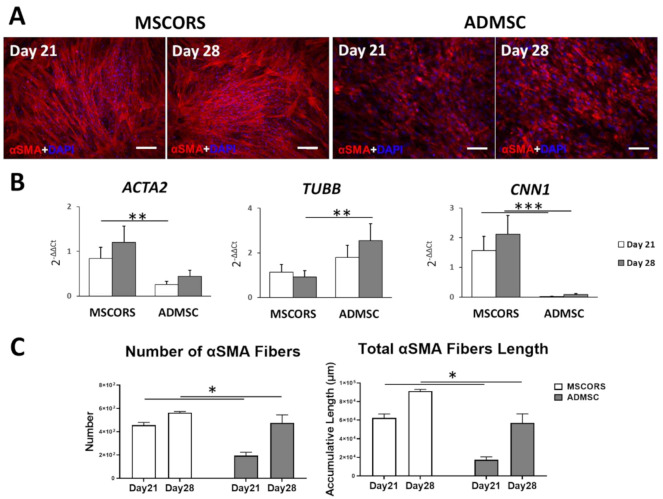
Smooth muscle differentiation of MSCORS and ADMSCs in vitro. ADMSCs and MSCORS underwent differentiation towards smooth muscle cell lineage for 21 and 28 days, evaluated via alpha smooth muscle actin (αSMA) immunostaining, qRT-PCR of smooth muscle marker gene expression and quantitative analysis of αSMA fibers. (**A**) Immunostaining of αSMA on ADMSC and MSCORS during smooth muscle cell differentiation (scale bar: 200 μm; magnification 10×). (**B**) Gene expressions of smooth muscle markers actin alpha 2 (*ACTA2)*, Tubulin Beta Chain *(TUBB)*, Calponin 1 *(CNN1)* in differentiated ADMSC and MSCORS. Gene expressions were normalized to the housekeeping gene HTRP-1 and calculated using 2^-ΔΔCt^ method against undifferentiated ADMSCs and MSCORS. (**C**) Quantitative analysis of αSMA fibers, in terms of number and length obtained using ImageJ software. Results are shown as mean ± SD (* *p* < 0.05, ** *p* < 0.01, *** *p* < 0.005).

Smooth muscle differentiation was assessed by αSMA protein-immunostaining and gene expression of αSMA (*ACTA2*), β-Tubulin (*TUBB*) and Calponin 1 (*CNN1*) by qRT-PCR (Figure 3). Smooth muscle cells derived from MSCORS showed elongated and fused morphology, with coalesced and multinucleated cell figures. These were characterized by prominent staining of αSMA in the αSMA-containing stress fibers, which was identical to the previously reported αSMA immunocytochemistry [23,26].

MSCORS-differentiated smooth muscle cells showed higher expression levels of smooth muscle markers than ADMSC cells in *ACTA2* and *CNN1* expressions.

Expression levels of *ACTA2*, *TUBB* and *CNN1* in both MSCORS and ADMSC were comparable to that of human aortic smooth muscle cells (HAoSMCs) used as positive control (Figure S2A). qRT-PCR gene expression analysis performed with 2^(−ΔΔCt)^ method indicated strong smooth muscle differentiation in MSCORS, and a higher expressions of *ACTA2* at day 21 (*p* = 0.024 < 0.05) than that observed in HAoSMCs, and a comparable expressions of *TUBB* and *CNN1* (Figure S2B).

Quantitative analysis of the αSMA actin stress fibers showed that both the number and length of the fibers in MSCORS-derived smooth muscle cells were significantly higher than in ADMSCs (*p* < 0.05).

### 3.4. MSCORS-Derived Endothelial Cells to Smooth Muscle Cell Sheet

**Figure 4:**
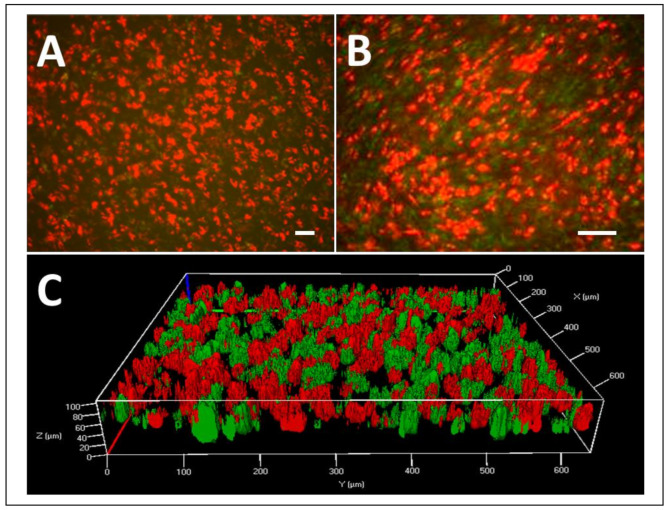
Differentiated endothelial cell attached to the smooth muscle sheet comprised of mesenchymal stem cells from hair follicle outer root sheath (MSCORS-SM-Sheet). Demonstration of the MSCORS derived endothelial cells attached to the MSCORS-based MSCORS-SM-Sheet labeled with fluorescent dyes. MSCORS-differentiated endothelial cells were labeled with a red fluorescent dye (PKH26), and the MSCORS-based smooth muscle sheet was labeled with a green fluorescent dye (PKH67). (**A**,**B**) Fluorescence microscopy of the endothelial cells (red) attached and layered atop the MSCORS-SM-Sheet (green) (scale bar 100 μm, magnification: (**A**) 10×, (**B**) 20×). (**C**) 3D reconstruction of the endothelial cell + smooth muscle sheet using laser scanning confocal imaging.

To explore the possibility of reconstructing tissue-engineered blood vessel using MSCORS in vitro, PKH26-labeled endothelial cells differentiated from MSCORS (red fluorescence) were seeded and left to attach to PKH67-labeled MSCORS-SM-Sheet (green fluorescence) (Figure 4A). Confocal imaging (Figure 4B) showed endothelial cells attached to the MSCORS-SM-Sheet, hereby displaying an alteration of cell morphology from spherical, qualifying as unattached, to irregularly shaped, which is suggestive of cell attachment. The 3D reconstruction of the cell-membrane complex (Figure 4C) showed that the endothelial cells were located chiefly atop the MSCORS-SM-Sheet, with partial integration into the ridges between large smooth muscle cells exhibiting an overlapping signal readout.

## 4. Discussion

In this study, we explored the differentiation potential of MSCORS towards endothelial and vascular smooth muscle, the two key cellular components of blood vessels and a base for neovascularization, hereby addressing their potential for vascular regeneration. Autologous MSCORS were obtained non-invasively by plucking hair follicles from a patient’s intact temporal scalp tissue, qualifying them as a preferable base for personalized regenerative therapies. Next to their notable isolation efficiency and high cell viability, MSCORS also exhibited high potential for osteo-, chondro-, adipo-, endothelial and smooth muscle differentiation [23]. Here, we investigated MSCORS as an autologous source of angiogenic progenitor “seed cells”, looking at their capability to deposit their own extracellular matrix. Furthermore, we analyzed the features of endothelial and smooth muscle cells differentiated from MSCORS and their behavior in co-culture, as a hallway towards future tissue engineering procedures.

All of the endothelial cells, except for the corneal endothelium, are derived and developed from the mesoderm during embryonic development [27], in line with the well-demonstrated MSC potential for endothelial differentiation [9,28,29]. VEGF and BMP-4 have been commonly used as effective mediators of endothelial differentiation in culture media. MSCORS and ADMSC were successfully differentiated towards the endothelial cell lineage, as shown at the level of gene expression, protein expression and endothelium functionality analysis. MSCORS exhibited a higher capacity for endothelial differentiation when compared to ADMSCs; this, coupled with the fact that ADMSCS are usually obtained via elective mid-grade invasive liposuction, qualifies MSCORS as a much preferable cell source for downstream applications. Moreover, MSCORS also showed higher ability to form a neo-angiogenic network than the ADMSCs, with higher grade of two-dimensional complexity. It is notable though that the subcellular localization of CD31 in MSCORS- and ADMSC-derived endothelial cells did not completely reflect that of the HUVEC, in particular at the level of cell junctions, implying that ex vivo isolation and in vitro culture and differentiation do impose developmental limitations. This may call for further optimization of the isolation, culture and differentiation procedures.

The MSCORS-derived endothelial cells expressed elevated level of *VEGF* gene compared to the HUVEC both after 21 and 28 days of differentiation, hereby displaying an internal production of VEGF and its increase during differentiation. This result is in line with previously reported evidence of intensive internal VEGF production after VEGF/BMP4-induced endothelial differentiation, with consequent implications for angiogenic cell therapy [30,31]. MSCORS-derived endothelial cells displayed clear expression of VEGF endothelial marker on gene and protein level.

It has been shown that the pre-existing endothelial progenitor cells residing in the bone marrow and peripheral blood can be mobilized to ischemic lesion areas, and facilitate the sprouting and anastomosis process during in vivo vascularization [32]. The ability of MSC-derived endothelial cells to build anastomotic networks has also been previously reported in vitro with tube-forming assay, confirming their angiogenic capacity [33] and in agreement with our quantification of MSCORS angiogenic network. Furthermore, MSCORS have hereby exhibited an ability to build anastomotic tubular networks clearly superior to those of ADMSCs. Taken together with the marker expression profile of MSCORS, they present a good base for further neoangiogenic development, which potentially has its place in bone repair and many other tissue engineering avenues.

In vitro differentiation of vascular smooth muscle cells has been achieved from various stem cell sources to date, including embryonic stem cells (hESC) [34], induced pluripotent stem cells (iPSC) [35], mesenchymoangioblasts [36], adipose MSCs [37] and bone marrow MSCs [38]. Differentiation of MSCs into smooth muscle cells is also a common feature of MSCs, even though it is not classically listed among the three basic MSC differentiation lineage capacities, serving as one of the classification criteria proposed by the International Society for Cell Therapy (ISCT) [39]. Vascular SMCs are found in bone marrow, defined as mesenchymal progenitor cells, and they are related to MSCs by origin and function [40]. In this study, we used TGF-β1, a commonly employed induction mediator for smooth muscle differentiation, to differentiate MSCORS. Historically, TGF-β1 has induced increased levels of αSMA expression and cell-mediated contraction in human bone marrow MSCs, as well as increased expression of αSMA in human adipose MSCs [41], therefore presenting a clearly measurable criterion for SMC differentiation. Likewise, we used this criterion to characterize the MSCORS-derived SMCs.

MSCORS differentiated into SMCs very efficiently. The expression of the αSMA was evident on both gene and protein level. Expression of other SMC-relevant genes such as *ACTA2* and *CNN1* was higher than in ADMSCs and comparable with the corresponding levels of native HAoSMCs, indicating the high differentiation potential of MSCORS. The end criterion of SMC differentiation, cumulative length of alpha-SMA, also quantitatively demonstrated higher level of SMC differentiation than in ADMSCs. These features, along with the fact that differentiated MSCORS present an easily up-scalable source of SMCs, places MSCORS among highly suitable cell sources for externally introduced SMC for purposes of regenerative angiogenesis.

Furthermore, MSCORS served not only as a base for deriving smooth muscle cells, but also for the culturing of a cell sheet made of cell-deposited extracellular matrix, and for co-culturing with endothelial cells, in turn pre-differentiated from MSCORS. Notably, the co-culture resulted in partially co-integrated layers of both endothelial cells and SMCs. Endothelial cells did not form a complete layer of endothelium atop the SMCs due to the cell seeding density and the large ridge size between smooth muscle cells. Importantly, MSCORS-derived endothelial cells attached to the smooth muscle cell sheet forming a co-cultured cell-membrane complex, suggesting they could be a valuable tool in tissue-engineered grafts.

Both MSCORS-derived endothelial cells and smooth muscle cells presented high pro-angiogenic potential in vitro that can be utilized in tissue-engineered repair, in all probability most efficiently if directly delivered as an integral part of a graft.

MSCORS offer several advantages compared to other MSC sources [22,23]. Firstly, they are obtained anytime by hair plucking, which is a completely non-invasive method. None of the other standardly used MSC sources can be obtained at a comparable level of invasiveness. MSCORS are also very easily handled: they can be quickly expanded, maintained in culture and cryopreserved for longer periods, without losing their differentiation potential and mobility, characteristics that they exhibit at a higher level in comparison to ADMSCs [23]. Moreover, cultured MSCORS quickly reach therapeutically relevant numbers. MSCs have previously been isolated from hair follicle outer root sheath [19,20,21]. Such procedures were either invasive since the hair follicles were obtained from the dissected scalp skin or they lacked reproducibility, scalability, purity and marker phenotyping, as analyzed and reported [22]. To the best of our knowledge, MSCORS isolation and culture procedure is the first one to provide all of the abovementioned aspects.

Herewith, MSCORS present a very good biological base for personalized treatments in regenerative medicine. Their differentiation capacity in vitro may bring along some limitations for in vivo and clinical uses, especially in complex tissues requiring intricated blood vessel networking, such as bone, or pancreatic islet [2,40,42]. Putative deposition of extracellular matrix and bone minerals, respectively, promise a good perspective for the effective engineering of said tissue as 3D in vitro constructs. The optimal ways of reaching a hi-fidelity anatomic structure and routes for a successful construct delivery remain to be explored further. 3D-printing of the cells into artificial extracellular matrices and an application as an integral part of grafts may present the most favorable options.

In conclusion, MSCORS showed superb endothelial and smooth muscle differentiation compared with ADMSC, suggesting a potential for vascularization and blood vessel engineering. This highly promising platform may provide future benefits in regenerative medicine.

## Data Availability

The data presented in this study are available on request from the corresponding author.

## Supplementary Materials

The following are available online at https://www.mdpi.com/2077-0383/10/5/911/s1, Figure S1: Endothelial differentiation and Tube Forming Assay of mesenchymal stem cells from hair follicle outer root sheath (MSCORS), adipose-derived mesenchymal stem cells (ADMSC) and human umbilical vein endothelial cells (HUVEC); Figure S2: Smooth Muscle differentiation of mesenchymal stem cells from hair follicle outer root sheath (MSCORS), adipose-derived mesenchymal stem cells (ADMSC) compared to human aortic smooth muscle cells (HAoSMC); Table S1: Medium Compositions; Table S2: Primer sequences.
