# Supplementary material for: The Angiogenic Potential of Mesenchymal Stem Cells from the Hair Follicle Outer Root Sheath
Source: J Clin Med. 2021 Feb 26;10(5):911. doi: 10.3390/jcm10050911 (PMC7956349; doi:10.3390/jcm10050911)
Supplement: Supplementary file 1 [file jcm-10-00911-s001.pdf]

Supplementary Data

Table S1. Medium Compositions.

Medium	Medium Composition
MSC Washing Medium	DMEM (Low Glucose)<br>100 U/ml Penicillin<br>100μg/ml Streptomycin<br>50 μg/ml Gentamycin<br>10 μg/ml Amphotericin B
MSCORS Isolation Medium	DMEM (Low Glucose)<br>10% Fetal Bovine Serum<br>1% ITS Premix<br>10ng/ml bFGF<br>20ng/ml rhEGF<br>2mM L-Glutamine<br>1% Pen/Strap (Penicillin 100U/ml, Streptomycin 100μg/ml)
MSC Cultivation Medium	DMEM (Low Glucose)<br>10% Fetal Bovine Serum<br>2mM L-Glutamine<br>1% Pen/Strap (Penicillin 100U/ml, Streptomycin 100μg/ml)
MSC Smooth Muscle Medium	DMEM (Low Glucose)<br>10% Fetal Bovine Serum<br>2mM L-Glutamine<br>10ng/mL TGFβ-1
MSC Endothelial Medium	DMEM (Low Glucose)<br>5% Fetal Bovine Serum<br>2mM L-Glutamine<br>0.05mM 2-Mercaptoethanol<br>30ng/ml VEGF<br>5ng/ml BMP-4
DPBST	DPBS<br>0.5% Tween 20

Table S2. Primer sequences.

Gene	NCBI Reference	Sequence (5'-> 3')		Tm	Location
CD31	NM_000442.5	Forward Primer	AACAGTGTTGACATGAA-GAGCC	59.12	298-319
		Reverse Primer	TGTAAAACAGCAC-GTCATCCTT	58.53	445-424
CD105	NM_001114753.2	Forward Primer	TGCACTTGGCCTACAATTCCA	60.2	765-785
		Reverse Primer	AGCTGCCCACTCAAGGATCT	60.91	871-852
VEGF	NM_001287044.1	Forward Primer	AGGGCAGAATCATCAC-GAAGT	59.44	150-170
		Reverse Primer	AGGGTCTCGATTGGATGGCA	60.98	224-205
VWF	NM_000552.4	Forward Primer	CCGATGCAGCCTTTTCGGA	60.75	354-372
		Reverse Primer	TCCCCAAGATACACGGA-GAGG	60.69	524-504
aSMA (ACTA2)	NM_001613.4	Forward Primer	AAAAGACAGCTAC-GTGGGTGA	59.59	219-239

		Reverse Primer	GCCATGTTCTATCGGG-TACTTC	58.61	294-273
CNN1	NM_001299.6	Forward Primer	GTCAACCCAAAATTGG-CACCA	59.86	308-328
		Reverse Primer	ACCTTGTTTCCTTTCGTCTTCG	59.13	490-469
β-Tublin (TUBB)	NM_178014.4	Forward Primer	AAGATCCGAGAAGAA-TACCCTGA	58.45	615-637
		Reverse Primer	CTACCAACTGATGGAC-GGAGA	58.9	735-715
HPRT1	NM_000194.3	Forward Primer	ACCACCGTGTGTTA-GAAAAGT	57.73	1195-1215
		Reverse Primer	CTGCTGACAAAGATTCAC-TGGT	58.86	1354-1333

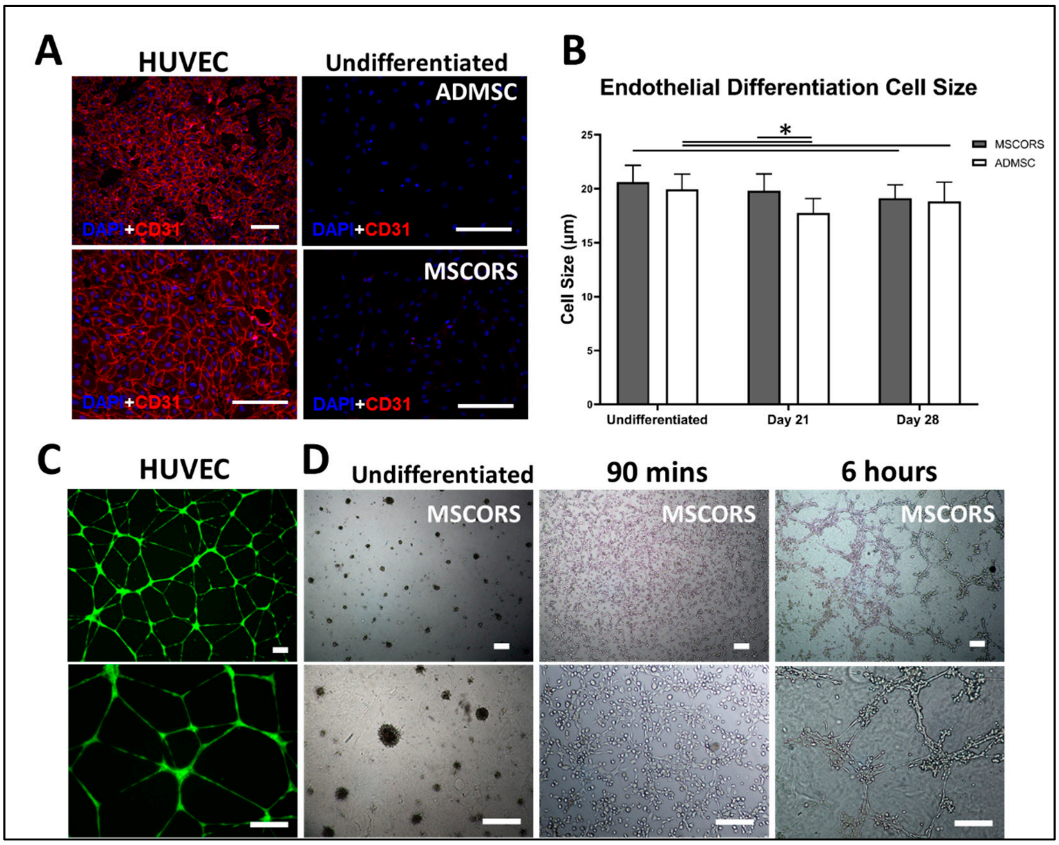

Figure S1. Endothelial differentiation and Tube Forming Assay of mesenchymal stem cells from hair follicle outer root sheath (MSCORS), adipose-derived mesenchymal stem cells (ADMSC) and human umbilical vein endothelial cells (HUVEC). (A) CD31 immunostaining of human umbilical vein endothelial cells (HUVEC) and undifferentiated controls of ADMSCs and MSCORS. (Scale bar 200 µm; magnification 10x and 20x). (B) Changes in cell sizes in ADMSCs and MSCORS after endothelial differentiation. Results are shown as mean±SD. (* $p < 0.05$). (C) Tube Forming Assay of HUVECS in the same condition as MSCORS and ADMSCs used as a positive control. Scale bar 200 µm; magnification 4x and 10x. (D) Anastomosis process of Tube Forming Assay in undifferentiated MSCORS and in differentiated MSCORS in a time-course of 90 min and 6 hours. Scale bar 200 µm; magnification 4x and 10x.

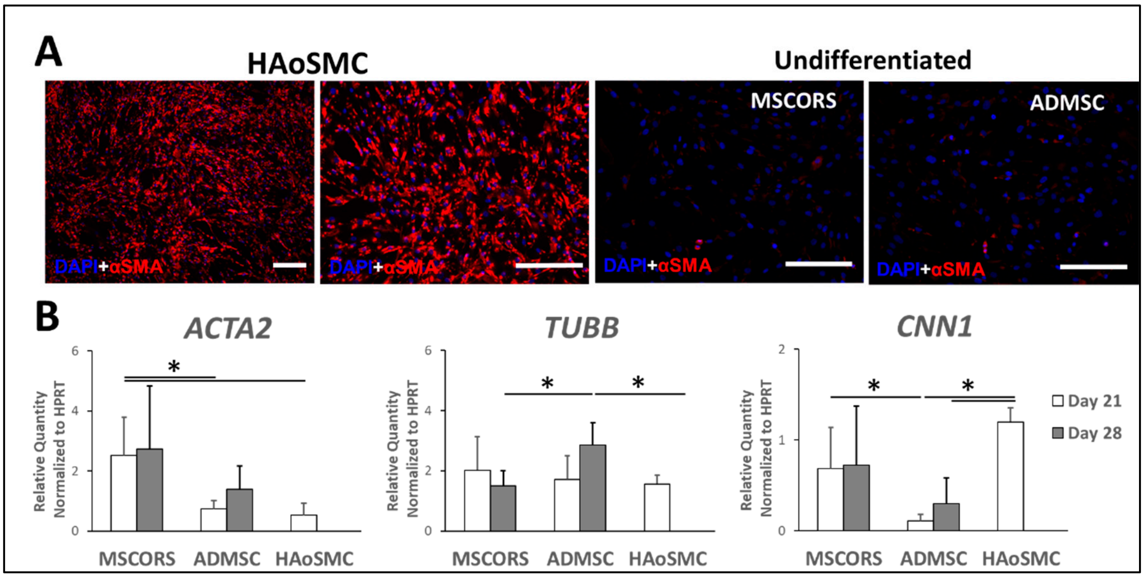

Figure S2. Smooth Muscle differentiation of mesenchymal stem cells from hair follicle outer root sheath (MSCORS), adipose-derived mesenchymal stem cells (ADMSC) compared to human aortic smooth muscle cells (HAoSMC). (A) Immunostaining of α-Smooth Muscle Actin (αSMA) on HA-oSMC and undifferentiated MSCORS and ADMSC. (B) Gene expressions of smooth muscle markers smooth muscle markers actin alpha 2 (*ACTA2*), Tubulin Beta Chain *(TUBB)*, Calponin 1 *(CNN1)* in differentiated MSCORS and ADMSC compared with HAoSMC. The results of qRT-PCR were analyzed using $2^{-\Delta\Delta Ct}$ method normalized to the housekeeping gene HTRP-1. Results were showed as mean±SD(* $p < 0.05$). Scale bar 200 µm; magnification (A) 10x and 20x.
